# Usefulness of Measuring Thiopurine Metabolites in Children with Inflammatory Bowel Disease and Autoimmunological Hepatitis, Treated with Azathioprine

**DOI:** 10.1155/2021/9970019

**Published:** 2021-06-17

**Authors:** Katarzyna Bąk-Drabik, Piotr Adamczyk, Justyna Duda-Wrońska, Dominika Dąbrowska-Piechota, Anna Jarzumbek, Jarosław Kwiecień

**Affiliations:** ^1^Department of Pediatrics, Faculty of Medical Sciences in Zabrze, Medical University of Silesia, Katowice, Poland; ^2^Department of Pediatrics, Faculty of Medical Sciences in Katowice, Medical University of Silesia, Katowice, Poland; ^3^Faculty of Medical Sciences in Zabrze, Students Association, Medical University of Silesia, Katowice, Poland

## Abstract

**Introduction:**

Thiopurines, such as azathioprine (AZA) and 6-mercaptopurine (6-MP), are immunomodulatory agents, used for the maintenance of remission in children with inflammatory bowel disease (IBD)—Crohn's disease (CD) and ulcerative colitis (UC), as well as with autoimmunological hepatitis (AIH). Measurements of thiopurine metabolites may allow identifying patients at risk for toxicity and nonadherence. It can also provide an explanation for the ineffectiveness of the treatment, observed in some patients. *Patients and Methods*. A retrospective analysis was carried out of sixty-eight patients (thirty-six patients with CD, eighteen with UC, and fourteen with AIH), treated with AZA. Thiopurine metabolites, 6-thioguanine nucleotide (6-TGN) and 6-methylmercaptopurine (6-MMP), were assayed by high-performance liquid chromatography (HPLC), and the AZA dose was adjusted when 6-TGN concentration was known.

**Result:**

Only twenty-five (41%) children had therapeutic 6-TGN concentrations, ten (16%) subjects had suboptimal 6-TGN concentrations, and twenty-six subjects (43%) had 6-TGN concentrations above the recommended therapeutic range. 6-MMP was not above the therapeutic range in any case. Seven subjects revealed undetectable 6-TGN and 6-MMP levels, indicating nonadherence. The mean AZA dose after the 6-TGN concentration-related adjustment did not differ, in comparison to the initial dose, either in IBD or AIH groups. The mean AZA dose was lower in AIH than in IBD. The subjects with an optimal 6-TGN level presented with a higher ratio of remission (88%) than the under- or overdosed patients (60% and 69%), respectively (Chi − square test = 3.87, *p* < 0.05).

**Conclusion:**

Timely measurements of thiopurine metabolites can be a useful tool to identify nonadherent patients before a decision is taken to switch to another drug. We may also spot the patients who receive either too low or too high doses, compensating dose deviations in an appropriate way. The patients with optimal 6-TGN levels presented a higher percentage of remission than the under- or overdosed patients. In most patients, both initial and adjusted AZA doses, lower than suggested in guidelines, appeared to be sufficient to maintain remission.

## 1. Introduction

Immunosuppressants are crucial drugs for the treatment of autoimmune disorders, including autoimmune hepatitis (AIH) and inflammatory bowel diseases (IBD). Azathioprine (AZA) has been proven to be a suitable medication with regard to its efficacy and side-effect profile.

AZA was synthesized in 1957 as a derivative of 6-mercaptopurine (6-MP) but earlier, in 1951, George Herbert Hitchings and Gertrude Elion discovered 6-MP and thioguanine (TG) as a result of searching for antimetabolites of nucleic acid bases that could arrest cell proliferation [1]. Thiopurines are prodrugs, metabolised by, at least, four different pathways until the final molecules, called thioguanine nucleotides (TGN), are obtained [1].

**Figure 1:**
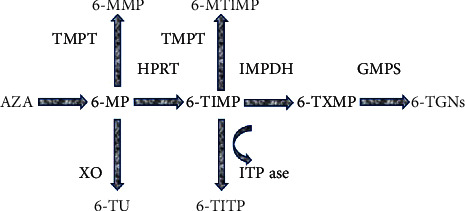
Azathioprine metabolism. AZA: azathioprine; HPRT: hypoxanthine phosphoribosyltransferase; IMPDH: inosine monophosphate dehydrogenase; GMPS: guanosine monophosphate synthase; ITPase: inosine triphosphatase; XO: xanthine oxidase; TPMT: thiopurine S-methyltransferase; 6-MP: 6-mercaptopurine; 6-MMP: 6-methylmercaptopurine; 6-MTIMP: 6-methylthioinosine monophosphate; 6-TXMP: 6-thioxanthylic acid; 6-TGNs: 6 thioguanine nucleotides; 6-TIMP: 6-thioinosine monophosphate; 6-TITP: 6-thioinosine triphosphate; 6-TU: 6-thiouric acid.

The metabolism of 6-MP involves three competing pathways: the first one being a degradation to thiouric acid (TUA), which is then excreted, the second one leads through methylation by thiopurine S-methyltransferase (TPMT) into 6-methylmercaptopurine (6-MMP), and the third one involves the breakdown of 6-MP into thioinosine monophosphate (TIMP), catalysed by hypoxanthine phosphoribosyltransferase (HPRT). TIMP is then further metabolised via inosine monophosphate dehydrogenase (IMPDH) into thioguanine triphosphate (TGMP). Kinases convert this into the TGNs [2]. TGNs are the active metabolites which exert immunomodulatory effects, whereas 6-MMP and 6-MMPR are the inactive and potentially toxic metabolites. These processes are presented in Figure 1.

AZA and 6-MP are immunosuppressants with short half-lives (3 and 1.5 hours, respectively) and, therefore, measuring their metabolites is a more appropriate method, both for adherence assessment and therapeutic drug monitoring. An intracellular accumulation of AZA/6-MP metabolites occurs over a period of 2–3 weeks [3]. Various studies have examined the relationship between 6-TGN levels in red blood cells and a clinical response to thiopurine therapy. There is an evidence that 6-TGN levels above 230 pmol/8 × 10^8^ erythrocytes correspond to a good clinical effect [4, 5], however, they do not guarantee remission. On the other hand, a 6-TGN level above 450 pmol/8 × 10^8^ erythrocytes may lead to an increased risk of myelotoxicity [6]. The concentrations of 6-MMP above 5400 pmol/8 × 10^8^ erythrocytes have been related to the development of hepatotoxicity [7]. Thiopurine metabolite measurements become more and more available, although their routine use is still limited by costs and technical requirements at laboratories. The primary aim of this study was to assess the usefulness of monitoring thiopurine metabolites in paediatric patients with IBD and AIH to assess their adherence to therapy and treatment safety. According to our knowledge, evaluating the use of metabolite measurement in children receiving thiopurine treatment was not performed in Polish children population. It is probably that the population from which the sample comes from could influence the results of the study. The secondary aim of the study was to compare AZA doses in both diseases and among the subgroups of patients, stratified according to the disease activity.

## 2. Material and Methods

### 2.1. Subjects

Sixty-eight children (thirty-one girls) with IBD and AIH, receiving azathioprine therapy in a consistent dose to maintain remission for at least 3 months, treated in one regional paediatric gastroenterology centre between April 2017 and May 2020, were identified by means of a retrospective review of their medical records. Within that group, there were thirty-six patients with CD, eighteen with UC, and fourteen with AIH. All the IBD children were treated according to the ECCO (European Crohn's and Colitis Organization) guidelines [8, 9], and the AIH children were treated according to ESPGHAN Hepatology Committee [10]. Seven patients with undetectable 6-TGN and 6-MMP levels were excluded from a detailed analysis concerning the assessment of the mean values of the AZA dose, initial and after correction, 6-TGN, 6-MMP, and a statistical analysis of those variables.

Among the remaining subjects, the following data were collected: demographics, body mass, type of disease, and laboratory data including white blood cell count, haemoglobin, aspartate aminotransferase (AST), alanine transaminase (ALT), and amylase and thiopurine metabolites (6-TGN, 6-MMP). IBD activity was determined, using the respective scales: PUCAI (the Paediatric Ulcerative Colitis Activity Index) and PCDAI (the Paediatric Crohn's Disease Activity Index). Biochemical remission in AIH was defined as a normalisation of transaminase activity and IgG concentration [10]. The mean duration of AZA therapy, before azathioprine metabolites were assayed, was 397 days (the range: 127-1294). The characteristics of the study group are presented in Table 1.

### 2.2. Methods

**Figure 2:**
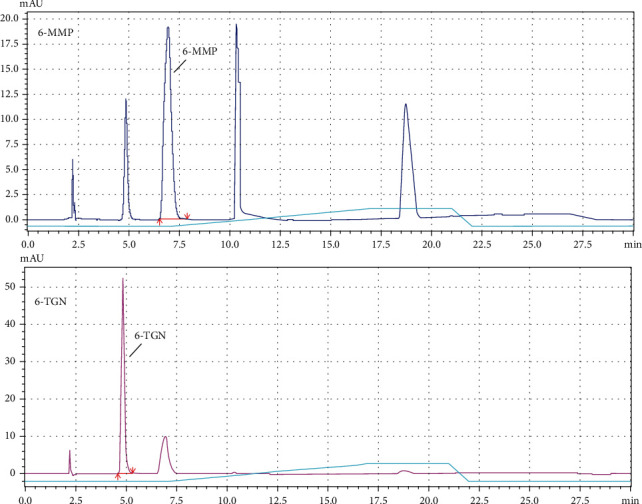
Representative examples of full HPLC spectra acquired for the measurements of thiopurine metabolites, 6-MMP (top) and 6-TGN (bottom). The marked peaks are specific for 6-MMP and 6-TGN, respectively. The remaining peaks do not have an influence on result interpretation.

Azathioprine metabolite (6-TGN and 6-MMP) levels were determined at an external analytical laboratory. In summary, cells, isolated from venous EDTA blood samples, were first three times washed with an isotonic buffer and then lysed, using the thermal disruption method. Subsequently, the lysates were deproteinised by incubation in acidic conditions and centrifuged for at least 15 min. at >10 000 rcf to remove cellular debris. The cleared lysates were analysed by high-performance liquid chromatography (HPLC) against a reversed-phase (RP) and by detection at 300-350 nm (using a UV-VIS detector). The obtained concentrations were quantified, using the AUC (area under curve) method, comparing the values against a standard curve, obtained with synthetic calibrators of known concentrations. Such raw reads were normalised, based on the RBC (red blood cell count) of each sample. The final results were calculated as pmol/8 × 10^8^ erythrocytes.  Figure 2 presents a typical spectrum, obtained for azathioprine metabolite measurement using HPLC resolved using water to methanol biphasic system.

### 2.3. Interpretation of the Results

The thresholds of 6-TGN and 6-MMP measurements and the interpretation of obtained results are presented in Table 2. Thus, it was aimed at keeping 6-TGN levels in the range of 230-450 pmol/8 × 10^8^ erythrocytes and 6-MMP below 5700 pmol/8 × 10^8^ erythrocytes.

## 3. Statistical Analysis

A statistical analysis was performed, using the Statistica software (StatSoft, Tulsa, OK, USA). Descriptive statistics for continuous variables were presented as mean values and standard deviations. The Shapiro-Wilk test was applied to verify the normality of data distribution. For a comparative analysis (comparisons between study groups), the applied statistical tools included the Student's *t*-test for independent samples or the Mann–Whitney *U*-test (for data with normal or abnormal distribution). The analysis of variance (ANOVA) with a post hoc least significance difference (LSD) test was used when more than 2 subgroups were compared. The longitudinal comparisons between the measurements, obtained at baseline and during follow-up, were assessed by the *t*-test for dependent samples or the Wilcoxon signed-rank test, whichever was appropriate, according to the data distribution. A correlation analysis was done by Pearson's or Spearman's correlation tests, whichever was appropriate, according to data distribution. Qualitative features were presented, juxtaposing the number of subjects with the percentage values in the defined subgroups. Comparisons of qualitative feature prevalence rates were performed by the Chi-square test. Significance for results in all the statistical analyses was assumed at *p* < 0.05.

The caregivers of the patients have consented to the use of their medical data in anonymised forms for statistical, educational, and scientific purposes, which is a standard procedure at the hospital. The current data analysis has been approved by the hospital authorities.

## 4. Results

### 4.1. Measurements of 6-TG and 6-MMP: An Interpretation of Metabolite Levels in the Group Receiving AZA

**Figure 3:**
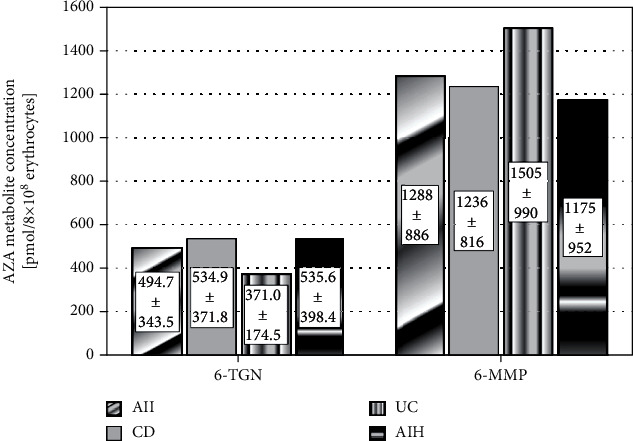
Interpretation of metabolite levels in the group receiving AZA. AZA: azathioprine; CD: Crohn's disease; UC: ulcerative colitis; AIH: autoimmunological hepatitis; 6-MMP: methylmercaptopurine; 6-TGN: 6-thioguanine.

The mean 6-TGN values in the whole group, as well as in CD, UC, and AIH patients, were 494.7 ± 343.5, 534.9 − ±371, 8 371.0 ± 174.5, and 535.6 ± 398.4 pmol/8 × 10^8^ erythrocytes, respectively. The mean 6-MMP values in the whole group, as well as in CD, UC, and AIH patients, were 1288 ± 886, 1236 ± 816, 1505 ± 990, and 1175 ± 952 pmol/8 × 10^8^ erythrocytes, respectively (see Figure 3).

Twenty-five (41%) children had therapeutic 6-TGN concentrations with a normal 6-MMP range. Ten (16%) had suboptimal 6-TGN concentrations with a normal 6-MMP range, which indicates that the AZA dose was below the therapeutic level. Twenty-six subjects (38%) had 6-TGN concentrations above the required therapeutic range with a normal 6-MMP concentration, which indicated hypomethylation and a potential toxicity for the bone marrow. Seven subjects had undetectable 6-TGN and 6-MMP levels, which indicated nonadherence to the therapy. 6-MMP concentrations above the range, which would indicate potential hepatotoxicity, were not identified in any case (see Table 3).

### 4.2. The Mean AZA Dose, Initial and after Adjustment

#### 4.2.1. The Difference between Pre- and Postadjustment of AZA Dose, Both in IBD (CD, CU) and AIH Subjects

**Figure 4:**
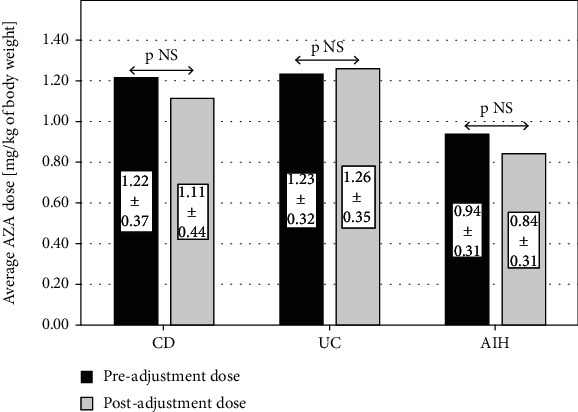
The difference between pre- and postadjustment AZA doses, both in IBD (CD, UC) and AIH subjects. AZA: azathioprine; CD: Crohn's disease; UC: ulcerative colitis; AIH: autoimmunological hepatitis.

The presented mean initial AZA dose in the whole study group, as well as in CD, UC, and AIH subgroups, was 1.15 ± 0.35, 1.22 ± 0.37, 1.23 ± 0.32, and 0.94 ± 0.30 (mg/kg/day), respectively. The dose after adjustment, based on 6-TGN concentrations, did not differ significantly from the initial AZA dose, either in the whole study group or in CD, UC, or AIH, and was 1.08 ± 0.44, 1.11 ± 0.4, 1.26 ± 0.32, and 0.84 ± 0.31 (mg/kg/day), respectively (see Figure 4). Neither was there any difference between the girls and the boys nor between the subgroups, defined according to the various methods of treatment or the disease activity, neither at baseline nor at the second assay.

#### 4.2.2. The Difference between IBD (CD, UC) and AIH Subjects, Both for Pre- and Postadjustment AZA Doses

A significant difference was revealed between IBD (CD, UC) and AIH subjects for the pre- and postadjustment AZA doses (*p* < 0.05), namely, the mean AZA dose was lower in AIH than in IBD patients. There was no correlation between the initial AZA dose and 6-TGN levels; after dose adjustment, based on 6-TGN concentrations that correlation could be clearly observed (*R* = −0.43, *p* < 0.005).

### 4.3. The Effect of Metabolite Measurements on Dose Modification in the Group Enrolled to the Study (*n* = 68)

**Figure 5:**
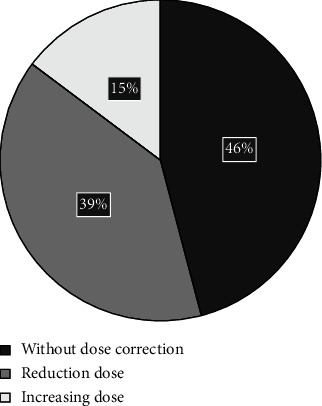
Therapeutic decision based on 6-TG concentrations. 6-TGN: 6-thioguanine.

In 7 cases, the level was undetectable and AZA was reintroduced. In 46% (28/61) cases, the dose was not changed. In the other cases (55%), the AZA dose was corrected. In 16% (10/61) cases, the AZA levels were below the range but the dose was increased only in 15% (9/61) subjects because of slightly decreased levels of leucocytes in the remaining patients of the study group. In 42% cases (26/61), 6-TGN-levels were above the range but dose modification was introduced only in 39% (24/61) subjects. In two patients, 6-TGN concentration was slightly above the range; the dose was maintained for the lack of remission (see Figure 5).

The subject with optimal 6-TGN levels presented a higher ratio of remission (88%) than those who were either under- or overdosed (60% and 69%), respectively (Chi − square test = 3.87, *p* < 0.05).

### 4.4. Adverse Outcomes

One patient (1.6%) developed leucopoenia (<3.5 WBCx × 10^9^), while none of the studied subjects developed any elevation of the liver enzymes. No other side effects, such as pancreatitis, glomerulonephritis, or lymphoma, were found in any of the patients throughout the study period.

### 4.5. A Correlation between 6-TGN Levels and Faecal Calprotectin

No correlation was found between faecal calprotectin and 6-TGN levels.

## 5. Discussion

Monitoring of thiopurine metabolites is a part of the safe treatment strategy and, together with other actions, such as the pretreatment screening for virus infections, a routine monitoring of leucocytes and aminotransferase, dose splitting strategies, allopurinol supplementation, and testing for TPMT deficiency, it helps reduce the risk of side-effects [11].

### 5.1. Nonadherence Rate

6-TGN levels are useful to identify nonadherence to thiopurine therapy, and it has been suggested that routine observations of the metabolites can help improve the adherence rates [12]. Several factors, such as sociodemographic, individual, family, disease regimen, and health care system, influence nonadherence. Indeed, in our study, only in 46% of cases, 6-TGN levels were within the therapeutic range and the dose was not changed. In that group of patients, the subjects with optimal 6-TGN levels presented higher a higher remission percent (88%) than those who were either under- or overdosed. That observation applied to all the AIH and IBD subjects. In a large study, where metabolite levels were reviewed in 9187 patients, the therapeutic goal was achieved only in 2444 patients (27%) [13].

In our study, seven subjects had undetectable 6-TGN and 6-MMP levels. A detailed medical history, regarding medicine intake regularity, revealed that those patients had not been taking the prescribed medicines for fear of side effects. In that group, there were five CD and two UC patients. We did not asses the adherence to treatment, using any specific questionnaires, although a multimethod assessment is more widely used [3]. In the future, we plan to assess adherence not only by objective methods but also with a specific questionnaire for both: parents and children. The lack of remission was an indication to consider the reintroduction of AZA therapy. In a Spanish study, the authors did not find any high rates of nonadherence (6.45%) but they strongly emphasised that the measurements of thiopurine metabolite concentrations could be useful to identify nonresponders before replacing or combining thiopurines with other alternative treatments (generally biological agents), with a consequent increase in both, a potential toxicity and costs [2]. Bokemeyer et al. [14] revealed in their study that, in a group of 65 adult CD patients, six (9.2%) had metabolite profiles that were indicative of nonadherence. The rate of nonadherence is comparable to the values in previously published studies [13, 15]. Hommel et al., evaluating adherence in 42 IBD adolescents, found that the majority of the sample (93%) had demonstrated quantifiable 6-TGN levels but only 14% were within the therapeutic range what indicated that nonadherence assessment was especially important in the group of adolescents, faced with learning to manage a chronic condition and negotiate normal developmental issues [16]. Alsous et al. [3], using a binary logistic regression analysis, identified the age to be independently predictive of adherence, with adolescents more likely to be classified as nonadherent. The mean age of our nonadherent subjects was 15 years. The patients, who are nonadherent, are more likely to have a more severe course of disease^,^ potentially necessitating the need for a more aggressive medical treatment, such as an increased corticosteroid use or surgery, present a higher risk of disease recurrence, in addition to these medical consequences, and, eventually, suffer of poor psychosocial functioning and low quality of life [17].

### 5.2. Underdosing

The regular measurements of metabolites can also identify patients who receive too low or too high drug dose, with available information about thiopurine methyltransferase (TPMT). In the Caucasian population, 0.3% subjects have TMPT deficiency, 6-11% have moderately reduced levels of TPMT activity, and 89-94% have normal TMPT activity. Tests for TPMT deficiency, prior to the onset of thiopurine therapy, should be the first step in personalising thiopurine therapy; however, cytopenia may still occur, despite normal TPMT activity, which does not identify patients at risk of other toxic or allergic adverse events, either. The latter information may help differentiate patients between those who have received a suboptimal AZA dose and those who have had higher TPMT activity, shifting AZA metabolism towards 6-MMP production. However, the cost and availability significantly reduce the use of the tests in routine practice. For this reason, we did not perform this test before the beginning of treatment at our hospital.

In our study, 10 cases (15%) had 6-TGN levels below the therapeutic range with 6-MMP within the range that indicated underdosing. It could also indicate irregular medication intake, so a detailed medical history is essential. 6-TG below the therapeutic range and 6-MMP above the range could indicate preferential metabolism via the TPMT pathway but it was not observed in our study. Another study showed even a higher percentage of underdosing [46].

It is recommended to keep 6-TGN levels between 250 and 450 pmol to maintain remission in inflammatory bowel disease [8, 9, 18, 19]. In one of the recent studies, it was revealed that serial thiopurine metabolite level assessments and dose adjustment aiming to maintain higher 6-TGN levels could be helpful to improve long-term outcomes in patients with IBD. The median 6-TGN levels were significantly higher in the patients who did not relapse, as compared with the levels in those patients who did relapse (233 vs. 167 pmol per 8 × 10^8^ erythrocytes, *p* = 0.025) [5]. Dubinsky et al. demonstrated that, in paediatric patients, 6 − TGN level ≥ 235 pmol per 8 × 10^8^ erythrocytes was associated with a therapeutic response to 6-MP [20]. Wright et al. also revealed that those patients, who developed active disease, accumulated significantly lower 6-TGN concentrations than those who remained in remission (175 vs. 236 pmol per 8 × 10^8^ erythrocytes, respectively). This study shows additionally that, due to intrapatient variability in 6-TGN production and the high incidence of compliance problems, a single 6-TGN reading may not be reflective of drug metabolism and serial measurements could be more useful [15].

All our children, whose 6-TGN level was below the therapeutic range, were IBD patients. They reported a regular intake of AZA, so AZA dose was increased. An ideal therapeutic 6-TGN-level for AIH was not determined [21]. The abovementioned Sheiko study revealed that 87% of 66 children maintained sustained biochemical remission in association with low 6-TGN levels, ranging from 50 to 250 pmol [21]. In a French study [22], the subjects in remission had similar-6-TGN levels (mean 6-TGN 436 pmol) as those with active disease (mean 6-TGN 406 pmol), which demonstrated the lack of correlation between 6-TGN levels and remission induction. After dose modification, follow-up measurements were carried out after three months.

### 5.3. Overdosing

In 39%, 6-TGN levels were above the range, with 6-MMP levels within the range, which indicated a potential TPMT deficiency and potential bone marrow toxicity. We did not observe 6 − TGN > 1000 pmol per 8 × 10^8^ erythrocytes with undetectable 6-MMP, which could suggest TPMT absence. A high concentration of 6-TGN is associated with an increased occurrence of adverse events. In a study by Lee et al. [23], the occurrence of leucocytopaenia and lymphopenia was associated with high concentrations of 6-TGN. Also, Pavlovska et al. showed similar results [24]. However, we did not observe this correlation in our study; the possibility of serious side effects should be considered in case of high 6-TGN levels. An interpretation of the range as high (>450 pmol per 8 × 10^8^ erythrocytes) depends on clinical features. In cases of active diseases, high 6-TGN levels suggest a thiopurine refractory case, prompting for an alternative treatment [25]. In case of remission or mild disease, dose reduction should be considered. In our study, dose modification was decided in 24 patients (39%), and in 2 patients, 6-TGN concentration was only slightly above the range, so the dose was maintained.

As in some other studies, we did not find any correlation between thiopurine dose and 6-TGN levels; therefore, increasing the drug dose may not be sufficient to reach the desired 6-TGN target [24, 26]. This may be explained by an increased methylation of intermediate 6-MP metabolites by inherited high levels of TPMT activity [5]. Other explanations refer to changes in azathioprine absorption, depending on disease activity, AZA formulation, or interactions with other drugs, such as mesalazine or and sulphasalazine [27]. On the contrary, Lee et al. found a positive correlation between the dose of AZA and the concentrations of 6-TGN (*p* < 0.0001) [23]. Despite the trend, favouring individualised dosing, other studies show no statistically significant differences in treatment efficacy between individualised dosing, based on baseline TPMT activity, and dosing, subsequently adjusted, according to the 6-TGN concentrations and weight-based AZA dosing [28].

### 5.4. Mean AZA Dose

In our study, the mean initial AZA dose and the dose after adjustment, based on 6-TGN concentrations, were lower than those, proposed in ECCO and ESPGHAN guidelines, both in IBD and AIH patients.

In AIH patients, the initial AZA dose was 0.5 mg/kg/day and then it was increased up to a maximum of 2.0-2.5 mg/kg/day. Our observation was similar to that in another study. Sheiko et al. [21] revealed that AZA dose of approximately 1.2–1.6 mg/kg/day was sufficient to maintain biochemical remission in the majority of patients. There was no correlation between AZA dose and 6-TGN levels, which was a similar conclusion to that in our study. The AZA dose in AIH was significantly lower, not only than proposed by ESPGHAN but also than that in IBD subjects. Those observations are coherent with Sheiko observation [22].

In inflammatory bowel diseases (CD and UC), the recommended dose is 2.0–2.5 mg/kg, and for its prodrug, 6-mercaptopurine, 1.0–1.5 mg/kg once daily. Our study revealed that the mean initial AZA dose in Crohn's disease and after modification was also lower than recommended. Neither was there any difference between the initial AZA dose and the dose after adjustment, based on 6-TGN concentrations, most likely for low AZA dose at baseline. The decision about the starting dose was made individually by a gastroenterologist. Various practical approaches among practitioners included thiopurine dosage, decisions about continuing thiopurines, and timing of metabolite assays. The other reason for lower AZA doses was the fact that, according to the previous studies, a lower dose of azathioprine is effective to induce and maintain remission in active Crohn's disease. Qian et al., in a prospective observational study, revealed that azathioprine,1.5 mg/kg/d, combined with steroids was as effective as AZA 2.0 mg/kg/d to induce remission of active CD in the first 6 months and to maintain remission of inactive CD in the first 2 years, without higher recurrence rate of active CD [29]. Another Chinese study confirmed that observation [30].

The mean AZA dose in CU patients, both at the beginning and after modification, was also lower than recommended (0.97, 0.87, and 2.0–2.5 mg/kg/day, respectively). The mean 6-TGN concentration was within the range (349, 47, 11 pmol/8 × 108 erythrocytes). Hibi et al. revealed the same observation in adults [31]. Walker et al. [26] also showed that, in the paediatric IBD children, the AZA dose, sufficient to maintain remission, was 1.3 ± 0.4 mg/kg; after excluding children on biologics, the effective azathioprine dose was 1.4 ± 0.5 mg/kg. Apart from receiving low AZA doses, 75% of IBD patients in our study were in remission. Those results suggest that therapeutic thiopurine metabolites can be achieved with a dose lower than recommended, although, since TMPT activity was not determined at the beginning of treatment, we could not know then that lower AZA doses could be sufficient to maintain remission.

### 5.5. Side Effects

Myelotoxicity is one of the most serious thiopurine-induced side effects and may occur at any time during the treatment. It is strongly linked to low TPMT enzyme activity and high 6-TGN blood levels. Myelotoxicity may also occur with normal TPMT activity, necessitating regular full blood count monitoring in clinical practice. In a review of 66 studies, including more than 8,000 thiopurine-treated patients, the incidence rate of drug-induced myelotoxicity was 3% per patient year of treatment [32]. In our study, only one patient (1.6%) developed leukopenia (<3.5 WBCx × 10^9^), but it was not related to high 6-TG levels. None of the children developed azathioprine toxicity, as defined by abnormal liver function tests. That observation was similar to that in another study [26]. On the contrary, Pavlovska et al. reported a higher percentage of adverse effects [24]. The most common treatment-related complication was leucocytopaenia (42.9%), followed by elevated transaminase levels (28.6%), aphthous ulcers (14.3%), and elevated amylase in serum (14.3%).

We did not find any correlation between faecal calprotectin (FC) and 6 T-GN levels. On the contrary, another study showed that, in patients with CD on AZA monotherapy, 6-TGN concentrations within a defined range (250–450 pmol/8 × 10^8^ erythrocytes) were associated with significantly lower FC [33].

We acknowledge the limitations of this study, including the lack of TPMT activity tests, justified by cost and availability issues, the sample size, and the retrospective design.

Measurements of 6-TGN and 6-MMP levels in IBD and AIH patients on AZA/6-MP may help identify patients at risk for toxicity and provide an explanation for the ineffectiveness of the treatment, observed in some patients. Thiopurine metabolite measurements become more and more available, although their routine use is still limited by costs and laboratory gear.

In conclusion, timely measurements of thiopurine metabolites can be a useful tool for the identification of nonadherent patients before adding or switching to another drug. This method can also identify patients receiving too low or too high doses, enabling subsequent corrections of drug doses, as the patients with optimal 6-TGN levels presented a higher percentage of remission than those who were under- or overdosed.

## Figures and Tables

**Table 1:** Background information about the group receiving azathioprine.

Characteristics	Values
Total number of patients	61
Females (percent)	27 (44%)
Age (years ± SD)	14.97 ± 2.6
Crohn's disease (CD)	31 (50%)
Ulcerative colitis (UC)	16 (26%)
Autoimmune hepatitis (AIH)	14 (23%)
Weight SDS (mean ± SD)	0.13 ± 1.11
CD	−0.23 ± 1.0
UC	−0.39 ± 0.7
AIH	0.53 ± 0.8
Height SDS (mean ± SD)	−0.43 ± 0.8
CD	−0.54 ± 0.9
UC	−0.60 ± 0.7
AIH	0.11 ± 0.7
BMI SDS (mean ± SD)	0.06 ± 0.9
CD	−0.04 ± 0.9
UC	−0.12 ± 0.8
AIH	−0.03 ± 2.7
Premonitoring azathioprine dose mg/kg (mean ± SD)	1.15 ± 0.3
CD	1.22 ± 0.3
UC	1.23 ± 0.3
AIH	0.94 ± 0.3
Postmonitoring azathioprine dose mg/kg (mean ± SD)	1.08 ± 0.4
CD	1.11 ± 0.4
UC	1.26.±0.3
AIH	0.84 ± 0.3
6-TGN (mean ± SD)	494.7 ± 345.3
CD	534.9 ± 371.8
UC	371.0 ± 174.5
AIH	535.6 ± 398.4
6-MMP (mean ± SD)	1288 ± 886.0
CD	1236 ± 816
UC	1505 ± 990
AIH	1175 ± 952
Disease activity:	
Remission/mild form of IBD	32 (68%)
Moderate form of IBD	10 (21%)
Severe form of IBD	5 (11%)
Remission of AIH	14 (100%)

SD: standard deviation; 6-MMP: 6-methylmercaptopurine; 6-TGN: 6-thioguanine; IBD: inflammatory bowel disease; AIH: autoimmunological hepatitis; CD: Crohn's disease; UC: ulcerative colitis.

**Table 2:** Interpretation of metabolite levels (measured in pmol/8 × 10^8^ erythrocytes) and recommended approaches. TPMT: thiopurine methyltransferase; 6-MMP: 6-methylmercaptopurine; 6-TGN: 6-thioguanine.

6-TGN	6-MMP	Interpretation	Recommendation
Very low	Very low	Nonadherence	Improve adherence
Low (<230)	Normal (<5700)	Insufficient dose	Consider dose increase
Normal (230-450)	Normal (<5700)	Therapeutic optimum	Further monitoring of treatment
High (>450)	High (>5700)	Overdosing	Dose reduction
Low (<230)	High (>5700)	Hypermethylation, risk of hepatotoxicity	Consider changing treatment or adding allopurinol with low doses of AZA
High (>450)	Normal (<5700)	Potential TPMT deficiency, risk of myelotoxicity	Monitoring blood test, consider dose reduction
Normal (230-450)	High (>5700)	Hypomethylation, risk of hepatotoxicity	Monitoring liver enzymes, split or reduce the dose
>1000	Undetectable	Potential TMPT absence, lack of methylation, risk of acute toxicity	Discontinuation of treatment

**Table 3:** Metabolite levels. Mean standard deviations and the range of values of 6-thioguanine (6-TGN), 6-methylmercaptopurine (6-MMPN), measured in pmol/8 × 10^8^ erythrocytes.

6-TGN levels (all subjects)	6-MMPN levels (all subjects)	Interpretation
Mean level: 494 ± 343	1288 ± 886	
Within range 41% (25/61)	Within range	Therapeutic optimum
Below range 16% (10/61)	Within range	Insufficient dose
Above range 43%(26/61)	Within range	Potential TPMT deficiency (potential bone marrow toxicity)
Undetectable 7 subjects	Undetectable	Nonadherence

6-MMP: methylmercaptopurine; 6-TGN: 6-thioguanine; TPMT: thiopurine S-methyltransferase.

## Data Availability

Data are available on request through the authors themselves. Contact: bak-drabik@wp.pl
